# Exosome miRNA Expression in Umbilical Cord Blood of High-Parity Sows Regulates Their Reproductive Potential

**DOI:** 10.3390/ani12182456

**Published:** 2022-09-16

**Authors:** Qiang Pu, Jie Chai, Li Chen, Changbao Liu, Changfeng Yang, Yongfu Huang, Jia Luo

**Affiliations:** 1 College of Animal Science and Technology, Southwest University, Chongqing 400715, China; puqiang1987@163.com (Q.P.); liucbzzz@163.com (C.L.); ycf19981024@163.com (C.Y.); hyf65@163.com (Y.H.); 2 Chongqing Liujiu Animal Husbandry Technology Co., Ltd., Chongqing 409099, China; 3 Chongqing Academy of Animal Sciences, Chongqing 402460, China; jiechai91@163.com (J.C.); lichen5696@163.com (L.C.)

**Keywords:** exosome, miRNA, reproductive potential, umbilical cord blood, pig

## Abstract

**Simple Summary:**

Increasing female reproductive efficiency, including the litter size and weight of piglets, is one of the biggest challenges in the swine industry. During the gestation period, the umbilical cord facilitates placenta–fetal communication; thus, it plays an indispensable role in intrauterine embryonic development and fitness. Herein, we analyzed the molecular mechanism in declining reproductive potential in high-parity sows by assessing the changes in the umbilical cord blood. Firstly, the best reproductive performance was at parity 3–7, gradually decreasing after parity 8 and angiogenesis was repressed in high-parity sows. Moreover, exosomes derived from multiparous sows exhibited pro-angiogenesis properties but were diminished in exosomes derived from high-parity sows. Additionally, the angiogenesis of sows was significantly decreased, increasing the risk of disease with the increase in parity, greatly limiting the reproductive potential of the sows. At the same time, miR-188-5p expression may play an important role in regulating the lifespan and reproductive potential of sows. These findings demonstrated that miRNAs in umbilical cord blood exosomes play a central role in intrauterine development and suggested novel insights on reproductive potential, which provide a reference for increasing the sow reproductive efficiency.

**Abstract:**

The objective of modern pig breeding is to improve the genetic reproduction performance potential of sows, including the litter size and weight of piglets. During the gestation period, the umbilical cord facilitates placenta–fetal communication; thus, it plays an indispensable role in intrauterine embryonic development and fitness. Herein, we analyzed the molecular mechanism in declining reproductive potential in high-parity sows by assessing the changes in the umbilical cord blood. Firstly, we analyzed the reproductive characteristics data of sows, followed by histological analysis of the umbilical cord phenotype. Next, we evaluated the effect of umbilical cord blood exosomes (UCB-EXO) on angiogenesis. Finally, the miRNA expression in UCB-EXO from high-parity sows with poor reproductive performance (OS) and multiparous sows with excellent reproductive performance (MS) was assessed. Overall, the best reproductive performance was at parity 3–7, gradually decreasing after parity 8 and angiogenesis was repressed in OS. However, exosomes derived from MS (Exo-MS) exhibited pro-angiogenesis properties but were diminished in exosomes derived from OS (Exo-OS). Additionally, the angiogenesis of sows was significantly decreased, increasing the risk of disease with the increase in parity, greatly limiting the reproductive potential of the sows. At the same time, miR-188-5p expression in Exo-OS was significantly higher than in Exo-MS (*p* < 0.01), implying that it may play an important role in regulating the lifespan and reproductive potential of sows. These findings demonstrated that miRNAs in UCB-EXO play a central role in intrauterine development. Further, the findings suggest novel insights on reproductive potential, which provide a reference for increasing the sow reproductive efficiency.

## 1. Introduction

Increasing the female reproductive efficiency, critical in maximizing productivity, is one of the biggest challenges in the swine industry [1]. For example, genetic selection for reproductive traits in sows such as litter size and weight is challenging due to their low heritability. Increasing the litter size is one of the crucial breeding goals in producing pig dam lines [2], which positively influences the number of pigs produced [3]. Reproductive indicators, particularly prolificacy, depend on the sow breed, body condition, age, and parity [4]. The sow parity significantly influences the total number born (TNB), number of healthy piglets (NHP), number of stillbirths (NSB), and the gestation period of sows. For example, the litter size is increased from parities 1 to 2, with the highest reproductive performances of sows in parities 3 and 5, followed by a gradual decline after parity 7 [5,6].

In contrast, the NSB is higher in multiparous sows than in primiparous sows. Thus, the NSB is increased to some extent with the increase in sow parity. For example, the number of stillbirths in parity 9 is significantly higher than in parities 1 to 8. In addition, the litter weight of piglets born at parity 9 is significantly lower than that of piglets born at parities 2 to 7 [7]. Therefore, an analysis of the molecular mechanism to provide a scientific and reasonable parity structure can effectively enhance the production potential of sows, accelerate genetic progress, and improve the overall farm productivity and economics.

The gestation period is closely related to the litter traits of sows, including the birth weight and litter size. The umbilical cord is the physical link facilitating the placenta–fetal communication during the gestation period; thus, it plays an indispensable role in intrauterine embryonic development [8]. Additionally, the umbilical cord changes with respect to the physical dimensions and composition, reflecting the changes in the fetal cardiovascular system [9]. Exosomes are membranous vesicles (30–100 nm in diameter) released into the extracellular space by various cell types. They play a significant role in cell communication, processing metabolic waste, and transporting nutrients to the developing fetus across the placenta [10,11]. In addition, exosomes derived from the placenta and umbilical cord deliver a significant proportion of circulating miRNAs, contributing to maternal–fetal communication during pregnancy, a new possible mechanism involved in fetal development [12,13]. However, the relationship between the reproductive potential of sows and the umbilical cord and the role of miRNAs in umbilical cord blood exosomes (UCB-EXO) during utero-embryo development has not been explored.

Therefore, this study aimed to analyze the molecular mechanisms associated with umbilical cord blood (UCB) during intrauterine embryonic development, which regulate the reproductive potential of sows. Firstly, we analyzed the reproductive performance, including the litter size, weight, and stillbirth of different sow breeds and parities. The offspring of high-parity sows (OS) and the multiparous sows with the best reproductive performance (MS) were included in the study. Subsequently, the morphological and histological characteristics of the umbilical cords and the pro-angiogenesis functions of exosomes derived from UCB (UCB-EXO) were analyzed. Finally, we evaluated the exosome miRNA transcriptome profiles in the UCB of OS and MS. The results revealed that the highly and differentially expressed miRNAs were mainly involved in angiogenesis and disease risk, with miR-188-5p playing an important regulatory role in the reproductive potential of sows. The findings of this study strengthen the understanding of the role of miRNAs in UCB-EXO during pregnancy, while outlining a novel, prospective, and possible mechanism for regulating reproductive potential in sows.

## 2. Materials and Methods

### 2.1. Experimental Samples

The study was performed in accordance with the Regulations for the Administration of Affairs Concerning Experimental Animals (Ministry of Science and Technology, China; revised in June 2004). All the reproductive performance data, including the breeding performance (parities, total number born (TNB), birth litter weight (BLW), number of healthy piglets (NHP), intrauterine growth retardation (IUGR) and number of stillbirth (NSB)) records of Duroc, Yorkshire, and Landrace were collected at the Chongqing Liujiu Animal Science and Technology Co., LTD (Chongqing, China) from 2012 to 2022. All newborn Yorkshire piglets used for umbilical cords and UCB collection were naturally born without genetic defects and had a cleared genetic background, and an average gestational age of 112 ± 2.2 d. In addition, six Yorkshire litters were selected for UCB-EXO miRNA transcriptome analysis at parity 9 and 4, representing the offspring of OS and MS, respectively.

### 2.2. Sample Collection, Exosome Preparation and Histological Analysis

We selected six litters (three at the 9th and 4th parities, each). We readied concentration 15% EDTA anticoagulant solution, putting two drops in the 2 mL syringe every time before collecting umbilical cord blood. Immediately after birth, the piglets were put under a heat lamp and the umbilical cord was picked up. Another researcher simultaneously used a syringe to draw blood from the umbilical artery and vein. After, upside down to make them blending fully timely, packing in 2 mL centrifuge tube, 1 mL per tube. Finally, exosomes in UCB were isolated according to our previous study [8]. After 1:1 dilution of umbilical cord blood in PBS, cells and cell debris were removed by two-step centrifugation (4 °C, 2000× *g*, 30 min; 4 °C, 12,000× *g*, 45 min), followed by filtration through a 0.22 µm filter. Exosomes were precipitated by ultracentrifugation (4 °C, 160,000× *g*, 2 h) and resuspended in PBS to obtain UCB-EXO. The particle size of UCB-EXO was analyzed by nanoparticle tracking analysis (NTA), while their morphology was observed by transmission electron microscope. Further, flow cytometry (Accuri C6 flow cytomenter, BD, America) was used to analyze the exosomes marker proteins CD63 (CD63-Antibody-FITC BD 557288, BD, New York, America) and CD81(CD81-Antibody-FITC BD 551108, BD, New York, America). The exosomes used for RNA extraction were purified by Exosomes Isolation Reagent (Ribobio, Guangzhou, China). After the piglets were euthanized, the tissue samples of cerebrum, liver, cerebellum, heart were collected and quickly placed in liquid nitrogen solution for further analysis.

Exosomes in umbilical cord blood were utilized for RNA isolation, after which the RNA was used for library construction and q-PCR analysis. The collected umbilical cords were used for immunohistochemistry analysis. Collected tissues were washed three times with phosphate-buffered saline (PBS) to remove blood and then fixed in 4% paraformaldehyde solution. The fixed umbilical cord tissue was embedded in paraffin blocks and stained with hematoxylin and eosin [8].

### 2.3. RNA Isolation and qRT-PCR

The RNA isolation and qRT-PCR were isolated as described previously as follows. The total exosomal RNAs (including miRNAs) were extracted using the TRIzol LS reagent (Invitrogen, California, CA, USA) according to the manufacturer’s instructions. Gel electrophoresis and spectrophotometry (Thermo, Waltham, MA, USA) were used to estimate the quality and concentration of the extracted total RNA, respectively. According to the manufacturer’s instructions, reverse transcription of miRNAs was then performed using a commercial kit (TaKaRa, Da Lian, China). Next, quantitative real-time PCR (qRT-PCR) was performed on a Bio-Rad IQ5 system (Bio-Rad, Hercules, CA, USA) using the SYBR Premix Ex Taq kit. The amplification conditions were as follows: initial denaturation at 95 °C for 30 s, followed by 40 cycles of denaturation at 95 °C for 30 s, annealing at 60 °C for 40 s, and extension at 72 °C for 30 s. The forward primer of miRNAs was identical in sequence and length to the miRNA itself based on our sequencing results. The expression levels of individual miRNAs were normalized against miRNA U6. The relative expression of the miRNAs was calculated using the 2^−ΔΔCt^ method [8].

### 2.4. Small RNA Library Construction and Sequence Analysis

The small RNA libraries were constructed using samples from the three Yorkshire sow litters at parity 9 (OS) and 4 (MS) mixed separately as described previously [8]. The libraries were constructed using approximately 1 μg of the total RNA using the TruSeq Small RNA Sample Prep Kits (Illumina) according to the manufacturers’ protocol. Single-end sequencing (36 bp) was performed on an Illumina Hiseq 2500 at the LC-BIO (Hangzhou, China), following the manufacturers’ protocol. The raw reads were subjected to the Illumina pipeline filter (Solexa 0.3), followed by the removal of adapter dimers, poor quality sequences, low complexity, common RNA families (rRNA, tRNA, snRNA, and snoRNA), and repeats using ACGT101-miR (LC Sciences, Houston, TX, USA). Unique sequences (18–26 nt) were subsequently mapped to species-specific precursors in miRBase 21.0 by BLAST search to identify known miRNAs and novel 3p- and 5p-derived miRNAs. Length variation at both 3′- and 5′-ends and a single mismatch within the sequence were allowed in the alignment. Unique sequences mapping to specific species mature miRNAs in hairpin arms were identified as known miRNAs. The identified miRNAs were uploaded to the Gene Expression Omnibus (GEO) at the NCBI under Accession Number GSE209805.

### 2.5. miRNA Target Gene Prediction and Pathway Analysis

Two bioinformatic algorithms (TargetScan 7.2 and miRanda 3.3a) were used to identify the miRNA binding sites to predict genes targeted by the differentially expressed miRNAs. Then the data predicted by the two algorithms were combined to obtain the overlap. Finally, the GO terms and KEGG pathways of the differentially expressed miRNA targets were annotated [8].

### 2.6. Cell Culture, Cell Proliferation Assay, and Scratch Assay

Human umbilical vein endothelial cells (HUVECs) were obtained from the Medical College of Suzhou University (Suzhou, China). The cells were seeded in 96-well plates containing Dulbecco’s modified eagle medium (DMEM) (Hyclone, Los Angeles, CA, USA) supplemented with 10% foetal bovine serum (FBS) (GIBCO, Waltham, MA, USA) and incubated at 37 °C with 5% CO_2_. The medium was changed every two days, and the cells were passaged at 90% confluence using 0.25% trypsin (Solarbio, Beijing, China). Next, the cells were transfected with exosomes as described previously [8]. Cell proliferation was assessed using a Cell Counting kit 8 (DOJINDO, Kyushu Island, Japan) according to the manufacturer’s protocol. The cells were transfected with exosomes and subjected to scratch analysis. HUVECs monolayers were scratched with 200-μl pipette tip when the cells in the 12-well plate were grown to near confluence. The culture supernatant was then washed three times with PBS to remove non-adherent cells and cell debris. The HUVECs cells were then incubated in DMEM containing 100μg/mL exosomes or PBS for 24 h to allow cell recovery. In addition, images were captured at the same fields of view at 0 h and 24 h after scratching the monolayer. “Wound healing” was assessed by analyzing the images using ImageJ Pro 1.51.

### 2.7. Statistical Analysis

All data are presented as means ± SEM. Differences between OS and MS means were analyzed using a Student’s two-tailed *t*-test for independent samples. Values were considered statistically significant when the *p* values were less than 0.05.

## 3. Results

### 3.1. Reproductive Performance of Sows at Different Parities

**Figure 1:**
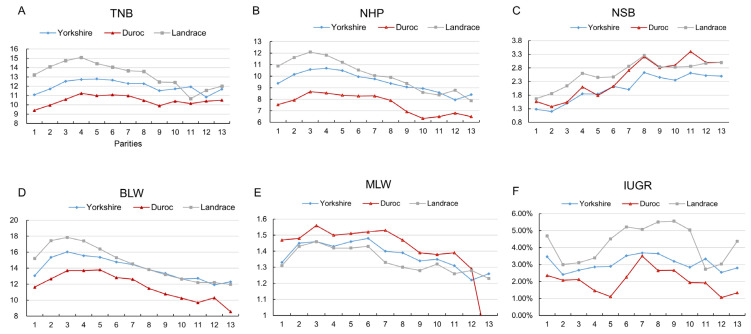
Reproductive performance of different parities sows. (**A**) TNB of different parities sows; (**B**) NHP of different parities sows; (**C**) NSB of different parities sows; (**D**) BLW of different parities sows; (**E**) MLW of different parities sows; (**F**) IUGR of different parities sows. Note: TNB: total number born; NHP: number of healthy piglets; BLW: birth litter weight; MLW: mean litter weight; NSB: number of stillbirth. IUGR: intrauterine growth retardation.

All the reproductive performance data, including the breeding performance records of Duroc (807 parities), Yorkshire (10,084 parities) and Landrace (28,898 parities) were collected for analysis. The TNB increased with an increase in sow parity. The Yorkshire breed reached the maximum TNB at parity 5 (12.78), followed by a slight decline, reaching the lowest TNB at parity 9 (11.53). On the other hand, Duroc reached the maximum TNB at parity 4 (11.24), then gradually decreased, reaching the lowest TNB at parity 9 (9.90). Similarly, Landrace reached its maximum TNB (15.09) at parity 4, then gradually decreased to its lowest TNB (10.67) at parity 11 (Figure 1A). At the same time, the NHP increased before parity 3 across the three breeds, then significantly decreased with an increase in parity (Figure 1B). On the contrary, the NSB increased with parity across the three breeds (Figure 1C). BLW and MLW also increased before the third parity, then significantly decreased at parities 6 and 8, respectively, across the three breeds (Figure 1D,E). In contrast, IUGR exhibited a decreasing trend from parities 1 to 3, then gradually increased, attaining a significant increase at parity 5, which was maintained until parity 10 (Figure 1F). Overall, parities 3 and 8 were the inflection points with significant changes in reproductive traits. Further analysis of the difference in reproductive performance of the three sow breeds at parities 1–2, 3–7, 8, and >8 revealed that TNB and NHP were significantly higher at parities 3–7 than 1–2 and were significantly decreased after parity 8. However, the IUGR at parity 8 in Yorkshire was significantly higher than in the other parities, with parity 3–7 being significantly higher than 1–2 and other parities > 8. Moreover, the NSB increased with an increase in parity, reaching the highest at parity 8, which was significantly higher than in the other parities. However, MLW found a significant increase only in Yorkshire parities 3–7. Overall, the reproductive performance gradually improved after parity 2, while the reproductive potential decreased after parity 8, with the best performance at parity 3–7 (Table 1).

### 3.2. OS Pigs Have Repressed Angiogenesis

**Figure 2:**
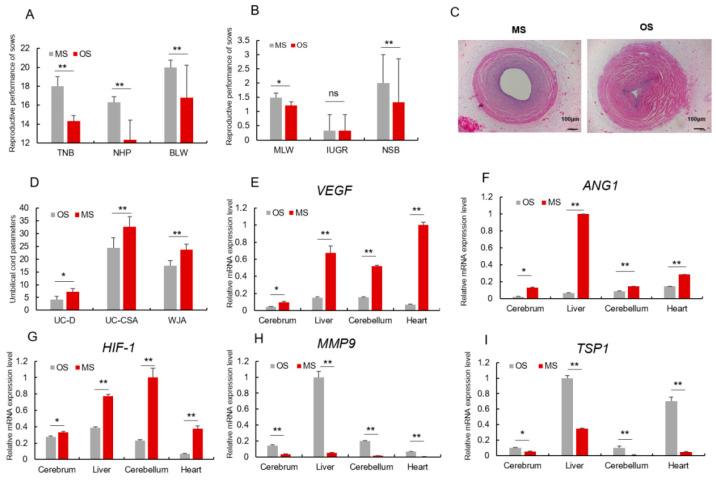
Angiogenesis of OS pig was repressed. (**A**,**B**) Reproductive performance of OS and MS sows. (**C**) The morphological characteristics of Umbilical Cord, 40×, scale = 100 μm, umbilical vein of MS; umbilical vein of OS. (**D**) Umbilical vein diameter (UV-D), umbilical vein cross-sectional area (UV-CSA), Wharton’s jelly (WJA). (**E**–**G**) mRNA relative expression level of pro-angiogenesis genes *(VEGF, ANG1, HIF-1*) in both MS and OS piglet’s tissues. (**H**,**I**) mRNA relative expression level of angiogenesis-inhibiting genes (*MMP9, TSP1*) in both MS and OS piglet’s tissues. * or ** represents significance at the 0.05 or 0.01 level, respectively, and ns represents no significance.

The average litter weights of OS and MS piglets at parities 9 and 4 were 1.31 ± 0.16 kg and 1.21 ± 0.14 kg, respectively. Their reproductive performance revealed that TNB, NHP, BLW, and MLW of OS sows were significantly lower than MS sows. However, the NSB in OS was lower than MS, while IUGR was not significantly different (Figure 2A,B). Fetal development and survival depend primarily on the functional integrity of the maternal-placental–fetal circulation. The changes of umbilical cord blood vessel morphology reflect the changes of placental blood vessel and placental microcirculation process. Moreover, the analysis of the physical parameters of the umbilical cord, including umbilical vein diameter (UV-D), and umbilical vein cross-sectional area (UV-CSA), revealed that they were significantly lower in OS piglets than MS piglets (Figure 2C,D). At the same time, the analysis of the expression of angiogenesis-related genes in key tissues (cerebrum, liver, cerebellum, heart) revealed that the pro-angiogenesis genes (*VEGF*, *ANG1* and *HIF-1*) were significantly down-regulated in OS, while the angiogenesis inhibiting genes (*MMP9* and *TSP1*) were significantly up-regulated (Figure 2E–G). Overall, angiogenesis was repressed in OS piglets.

### 3.3. The Pro-Angiogenesis Role of Exo-OS Was Repressed

**Figure 3:**
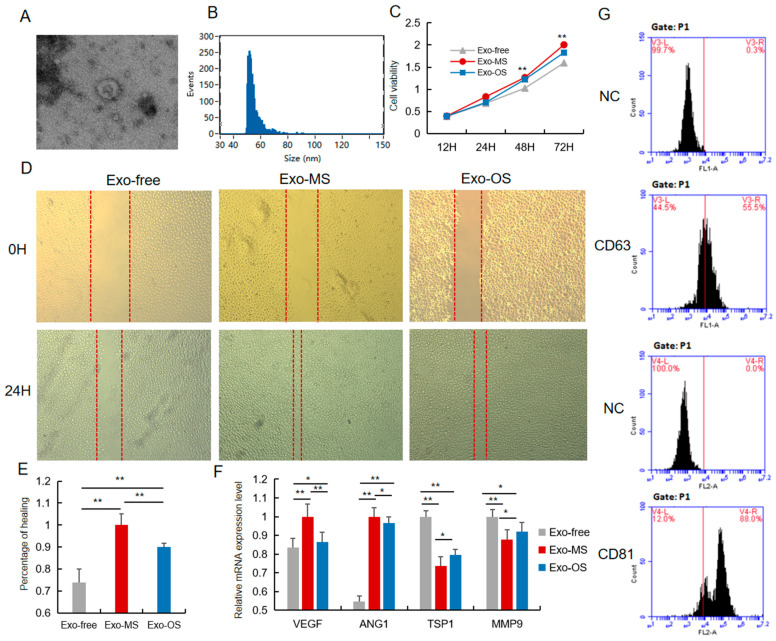
The pro-angiogenesis role of Exo-OS was repressed. (**A**) The 3D morphology of isolated pig UCB-EXO observed in electron microscope. (**B**) The line profile of electron microscope image size for UCB-EXO. X- and Y-axes are the size and events, respectively. (**C**) Cell viability was measured following Exo-OS, Exo-MS, or PBS treatment for 24 h, using the CCK8 analysis. (**D**) The migration of HUVEC cells in vitro after 24 h of treatment with Exo-MS, Exo-OS, or PBS was evaluated using scratch assay (*n* = 3). (**E**) Percentage of cells healed. (**F**) The expression level of *VEGF, ANG1, TSP1*, and *MMP9* mRNA after treating Exo-MS, Exo-OS, or PBS. (**G**) Positive rate of exosome marker proteins CD63 and CD81 detected by flow cytometry. * or ** represents significance at the 0.05 or 0.01 level, respectively.

The UCB contained numerous exosomes, measuring approximately 70 nm × 50 nm, appearing as flattened, donut-like structures (Figure 3A,B). Positive rates of exosomes marker proteins CD63 and CD81 were 55.5% and 88.0%, respectively (Figure 3G). To investigate the role of UCB-EXO in angiogenesis, HUVECs were analyzed. In addition, the role of exosomes derived from umbilical veins in pro-angiogenesis activities was explored. Although the umbilical vein is the main nutrient supply for the fetus, there is increasing evidence that the umbilical vein in neonatal pigs is hypoplastic. The UCB-EXO from MS sows (Exo-MS) and UCB-EXO from OS sows (Exo-OS) enhanced cell growth by increasing the HUVECs viability, although the facilitation effect of Exo-OS was significantly lower (Figure 3C). The scratch assay further revealed that Exo-MS and Exo-OS significantly promoted HUVEC migration, compared to EXO-free HUVECs. However, the migration of HUVECs treated with Exo-OS was significantly lower than HUVECs treated with Exo-MS (Figure 2D,E). Furthermore, the pro-angiogenesis genes *VEGF* and *ANG1* were up-regulated when cells were cultured in the presence of Exo-MS and Exo-OS, although the expression level with Exo-OS was significantly lower than with Exo-MS. Moreover, the anti-angiogenesis genes *TSP1* and *MMP9* were down-regulated in the presence of Exo-OS, although the expression level in the presence of Exo-OS was significantly higher than Exo-MS (Figure 2F). Overall, Exo-MS exhibited pro-angiogenesis properties higher than those of Exo-OS.

### 3.4. Enriched miRNAs in Exo-OS and Exo-MS

**Figure 4:**
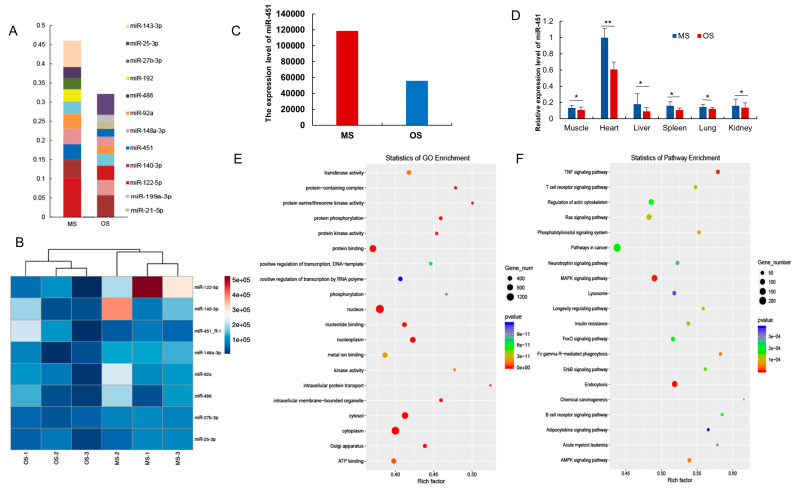
Analysis of pig cord blood enriched miRNAs of OS and MS. (**A**) Analysis of miRNAs generally abundant across Exo-OS and Exo-MS; (**B**) Clustering of differentially expressed miRNAs of Exo-OS and Exo-MS; (**C**) The expression level of miR-451; (**D**) Relative expression level of miR-451 in different tissues; (**E**) GO enrichment of high expressed miRNAs; (**F**) KEGG enrichment of high expressed miRNAs. * or ** represents significance at the 0.05 or 0.01 level, respectively.

A miRNA is a highly mobile epigenetic molecule that transmits information between the mother and the fetus through the UCB. Analysis of the differences in phenotypic traits between OS and MS in this study revealed a possibility of miRNA in UCB involvement in communication between the mother and fetus. The sequencing of Exo-OS and Exo-MS miRNAs revealed the enriched miRNAs (Top 10) in OS and MS were 31.21 and 46.06%, respectively (Figure 4A). Interestingly, the expression profile of the most abundant (top 10) and unique miRNAs had great similarities between OS and MS, consistent with the miRNA cluster analyses (Figure 4B). In addition, the analysis of the transcription characteristics of Exo-OS and Exo-MS miRNAs identified numerous highly expressed miRNAs involved in angiogenesis and immunity. Among them, miR-122-5p, a liver-specific microRNA involved in endothelial proliferation and angiogenesis, and miR-451, a blood-specific miRNA involved in angiogenesis, were highly expressed in OS and MS (Figure 4C). However, miR-451 was only highly expressed in major organ tissues, but down-regulated in OS, especially in the heart (Figure 4D), implying it plays important regulatory roles in the functional maintenance during organ development. The target genes for the top-ten-expressed miRNAs in OS and MS and their GO terms and KEGG pathways are presented in Figure 4E,F. The key GO term was the nucleus, cytoplasm, and transferase activity, while the key KEGG pathways were the Endocytosis, MAPK signaling pathway, and TNF signaling pathway, implying the miRNAs expressed in UCB-EXO played a central role in intrauterine development.

### 3.5. Functional Analysis of Differently-Expressed UCB-EXO miRNAs between OS and MS

**Figure 5:**
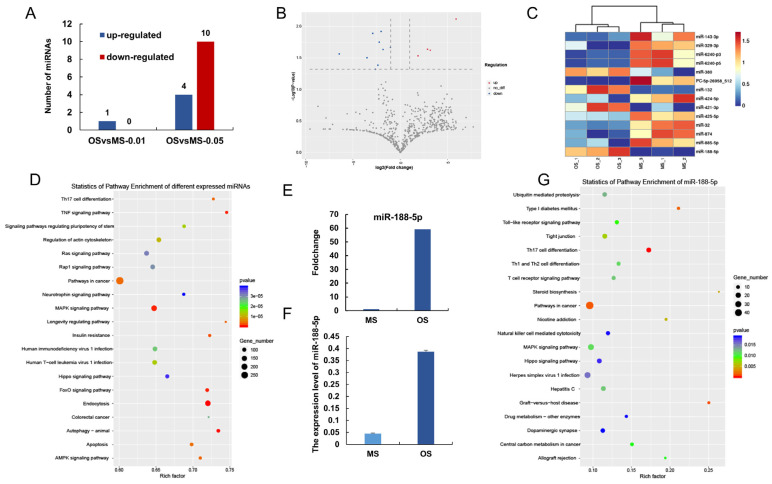
Functional Analysis of different-expressed UCB-EXO miRNAs between OS and MS. (**A**) Number of differentially expressed miRNAs between OS and MS; (**B**) Volcano of differentially expressed miRNAs between OS and MS; (**C**) Cluster analyses of differentially expressed miRNAs between OS and MS; (**D**) KEGG enrichment of different expressed miRNAs between OS and MS; (**E**) Foldchange of the miR-188-5p expression between OS and MS; (**F**) The expression level of miR-188-5p in OS and MS by q-PCR; (**G**) KEGG enrichment of miR-188-5p.

Analysis of the exosome miRNA transcriptome profiles of Exo-OS and Exo-MS identified 14 differentially expressed miRNAs between OS and MS (*p* < 0.05) (Figure 5A, B). The miRNA cluster analyses further revealed that OS and MS are separated (Figure 5C). The differently expressed miRNAs were mainly annotated to Endocytosis, Autophagy-animal, and TNF signaling pathways (Figure 5D). In addition, the expression of miR-188-5p in Exo-OS was significantly higher than in Exo-MS (*p* < 0.01; Figure 5E,F). Further analysis of its functional enrichment revealed that miR-188-5p was mainly annotated to Th17 cell differentiation and pathways in cancer and Type I diabetes mellitus. Overall, the miRNA in UCB-EXO of OS pigs regulated the health status of the sows by increasing the risk of disease.

## 4. Discussion

Maximizing the reproductive potential during the sows’ lifetime decreases the production costs and economic inefficiency in commercial breeding herds [14]. Low parity females, especially pregnant gilts, aged sows, and sows at parity 1 have lower reproductive performance than sows in parities 2–5 [6,15]. In this study, the reproduction performance records of Duroc (807 parities), Yorkshire (10,084 parities) and Landrace (28,898 parities) at parities 1–13 revealed that parities 3 and 8 were the inflection points of significant changes in reproductive traits. In addition, the reproductive performance gradually improved after parity 2, then decreased after parity 8, with the best performance at parities 3–7. The utilization life of sows is significantly prolonged after an extensive breeding period. However, fertility declines due to decreased ovulation and fertilization rates in aged sows. Moreover, with age, the sows have increased embryonic mortality/pregnancy loss and stillborn piglets due to the slower responses to the space demands by growing fetuses and stimuli from parturition processes [14].

The umbilical cord is the physical link between the placenta and the fetus and plays an indispensable role in the intrauterine development of the fetus during the embryonic period. There are many lean umbilical cords in intrauterine growth-restricted fetuses compared to appropriate-for-gestational-age fetuses [16], with the umbilical cord structure driving recurrent fetal death [17]. Exosomes and miRNAs have been found to play an important role in maternal–fetal interaction, such as exosomes in the porcine trophectoderm cells contain specific miRNAs, which induce the proliferation of the maternal endothelial cells and promote the angiogenic processes [18] at the maternal–fetal interface [19]. Additionally, miR-150 in the UCB-EXO of sows promotes angiogenesis [8]. In this study, we chose the UCB-EXO sample of high-parity sows with poor reproductive performance at the ninth parity (OS) and multiparous sows with excellent reproductive performance at the fourth parity (MS). Histological analysis of umbilical cord phenotype and the analysis of the effect of UCB-EXO on angiogenesis revealed that UV-D and UV-CSA of OS were significantly lower than MS, which is consistent with the previous report on the decrease in the umbilical cord of IUGR [20]. In addition, the pro-angiogenesis genes (*VEGFA, ANG1*, and *HIF-1*) were significantly down-regulated in OS, while the angiogenesis inhibiting genes (*MMP9* and *TSP1*) were significantly more up-regulated than in Exo-MS.

Moreover, Exo-MS exhibited pro-angiogenesis properties, markedly diminished in Exo-OS, as manifested in decreased cellular activity and reduced HUVECs migration. In addition, expression of the pro-angiogenesis genes *VEGF* and *ANG1* was down-regulated while that of the anti-angiogenesis genes *TSP1* and *MMP9* were more up-regulated than Exo-MS. Therefore, the repressed angiogenesis in OS was most likely due to the effect of UCB-EXO. Previous studies have found that placenta-derived exosomes induce vasculogenesis and angiogenesis through an oxygen-sensing mechanism [21]. Thus, exosomes in maternal and umbilical cord blood could regulate angiogenesis during pregnancy, a role played by the exosomal miRNAs, to maintain normal pregnancy [22].

Moreover, the analysis of the miRNA transcription characteristics of Exo-OS and Exo-MS revealed that most highly expressed miRNAs were related to angiogenesis and disease susceptibility. Among them, miR-122-5p, a liver-specific microRNA highly expressed in both OS and MS promoted endothelial proliferation and angiogenesis. miR-122-5p regulates cellular stress response, hepatocarcinogenesis, and hepatitis viruses. miR-122 is also a pro-inflammatory microRNA, which, when exported to tissue-resident macrophages, induces the expression of inflammatory cytokines in the tissue [23]. At the same time, miR-451, a blood-specific miRNA, involved in angiogenesis and necessary for erythropoiesis and erythroid homeostasis [24,25], was also highly expressed in OS and MS. Overall, the miRNAs expressed in UCB-EXO played a more central role in intrauterine development. However, restrained angiogenesis greatly limited the reproductive potential of sows. Still, miR-188-5p annotated to Th17 cell differentiation and pathways in cancer and Type I diabetes mellitus were significantly up-regulated in OS. miR-188-5p is commonly associated with tumor growth and metastasis [26,27,28]. miR-188-5p induces cell senescence and plays a key role in aging-related diseases [29,30]. Therefore, inhibition of miR-188-5p reduces the neovessel density and the infiltration of T cells and macrophages, inhibiting inflammatory responses [31]. Thus, miR-188-5p plays a significant role in the age-related reproductive performance decline of sows. Furthermore, angiogenesis significantly decreased, and disease risk increased with the increase in parity, which restricted the intrauterine development of fetuses, reducing the reproductive potential of sows.

## 5. Conclusions

Angiogenesis of OS is repressed during intrauterine embryonic development. Although UCB-EXO possesses pro-angiogenic characteristics, the effect is significantly reduced in Exo-OS. The disease risk in sows is increased in OS., and miRNAs in UCB-EXO play significant regulatory roles in important processes of embryo development and health, restricting the intrauterine development of fetuses and reducing their reproductive potential. miR-188-5p plays a significant role in the age-related reproductive performance decline of sows. Overall, the findings in this study provide new insights into reproductive potential, epigenetic phenomena, and directions to improve the reproductive performance of breeding pigs.

## Figures and Tables

**Table 1:** The difference of reproductive performance of sows at 1st–2nd, 3rd–7th, 8th and >8th parities.

	Landrace				Duroc				Yorkshire			
Parity	1–2	3–7	8	>8	1–2	3–7	8	>8	1–2	3–7	8	>8
Litters	671	1009	99	147	302	365	28	36	3396	4631	505	877
TNB	13.69 ± 3.33 ^a^	14.55 ± 3.64 ^b^	13.62 ± 3.25 ^a^	12.31 ± 3.65 ^c^	9.67 ± 2.71 ^a^	10.82 ± 2.67 ^b^	11.04 ± 2.74 ^b^	10.25 ± 1.73 ^a^	11.39 ± 3.28 ^a^	12.63 ± 3.65 ^b^	12.30 ± 3.71 ^b^	11.75 ± 3.61 ^a^
IUGR	0.53 ± 0.96 ^a^	0.60 ± 0.97 ^a^	0.56 ± 0.89 ^a^	0.65 ± 1.14 ^a^	0.16 ± 0.43 ^a^	0.21 ± 0.55 ^a^	0.07 ± 0.26 ^a^	0.17 ± 0.38 ^a^	0.34 ± 0.71 ^a^	0.38 ± 0.81 ^bc^	0.45 ± 0.87 ^b^	0.36 ± 0.73 ^ac^
NHP	11.32 ± 2.85 ^a^	11.45 ± 2.94 ^a^	9.75 ± 2.74 ^b^	8.96 ± 2.83 ^c^	7.95 ± 2.66 ^a^	8.61 ± 2.51 ^b^	7.71 ± 2.64 ^abc^	7.14 ± 1.81 ^ac^	9.76 ± 2.9 ^a^	10.37 ± 2.92 ^b^	9.5 ± 2.79 ^a^	8.92 ± 2.67 ^c^
NSB	1.76 ± 2.10 ^a^	2.46 ± 2.35 ^b^	3.26 ± 2.92 ^c^	2.65 ± 2.44 ^bc^	1.49 ± 1.67 ^a^	1.95 ± 1.92 ^b^	3.21 ± 3.01 ^c^	2.94 ± 1.74 ^c^	1.24 ± 1.56 ^a^	1.82 ± 2.00^b^	2.33 ± 2.13 ^c^	2.46 ± 2.30 ^c^
MLW	1.38 ± 0.27 ^a^	1.39 ± 0.25 ^a^	1.36 ± 0.31 ^a^	1.35 ± 0.30 ^a^	1.51 ± 0.30 ^a^	1.52 ± 0.30 ^a^	1.51 ± 0.38 ^a^	1.42 ± 0.42 ^a^	1.41 ± 0.24 ^a^	1.45 ± 0.25 ^b^	1.42 ± 0.28 ^a^	1.41 ± 0.28 ^a^

Note: TNB: Total number born; NHP: Number of healthy piglets; IUGR: Intrauterine growth retardation; LW: litter weight; MBW: mean birth weight; NSB: number of stillbirth; MLW: Mean litter weight. Same letters mean insignificant, different letters mean significant.

## Data Availability

Not applicable.
