# Correction: Pu et al. Exosome miRNA Expression in Umbilical Cord Blood of High-Parity Sows Regulates Their Reproductive Potential. *Animals* 2022, *12,* 2456

**DOI:** 10.3390/ani14020345

**Published:** 2024-01-22

**Authors:** Qiang Pu, Jie Chai, Li Chen, Changbao Liu, Changfeng Yang, Yongfu Huang, Jia Luo

**Affiliations:** 1 College of Animal Science and Technology, Southwest University, Chongqing 400715, China; puqiang1987@163.com (Q.P.); liucbzzz@163.com (C.L.); ycf19981024@163.com (C.Y.); hyf65@163.com (Y.H.); 2 Chongqing LiuJiu Animal Husbandry Technology Co., Ltd., Chongqing 409099, China; 3 Chongqing Academy of Animal Sciences, Chongqing 402460, China; jiechai91@163.com (J.C.); lichen5696@163.com (L.C.)

## Error in Figure

In the original publication [1], there was a mistake in “Figure 2C. The morphological characteristics of Umbilical Cord, and Figure 3D. The migration of HUVEC cells in vitro after 24 h of treatment with Exo-MS, Exo-OS, or PBS” as published. “This picture was improperly used due to a mistake in organizing pictures during the data handover process during the COVID-19 pandemic, but it does not affect the results and conclusions of the paper”. The corrected “Figure 2. Angiogenesis of OS pig was repressed, and Figure 3. The pro-angiogenesis role of Exo-OS was repressed.” appear below. The authors state that the scientific conclusions are unaffected. This correction was approved by the Academic Editor. The original publication has also been updated.

## Figures and Tables

**Figure 2:**
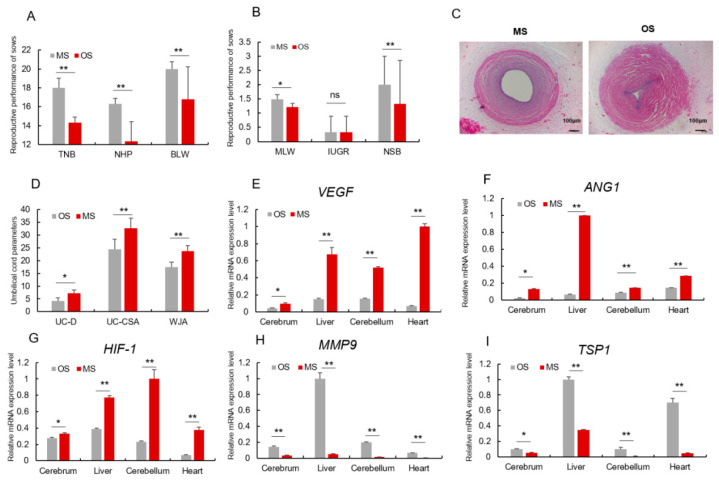
Angiogenesis of OS pig was repressed. (**A**,**B**) Reproductive performance of OS and MS sows. (**C**) The morphological characteristics of Umbilical Cord, 40×, scale = 100 μm, umbilical vein of MS; umbilical vein of OS. (**D**) Umbilical vein diameter (UV-D), umbilical vein cross-sectional area (UV-CSA), Wharton’s jelly (WJA). (**E**–**G**) mRNA relative expression level of pro-angiogenesis genes *(VEGF, ANG1, HIF-1*) in both MS and OS piglet’s tissues. (**H**,**I**) mRNA relative expression level of angiogenesis-inhibiting genes (*MMP9, TSP1*) in both MS and OS piglet’s tissues. * or ** represents significance at the 0.05 or 0.01 level, respectively, and ns represents no significance.

**Figure 3:**
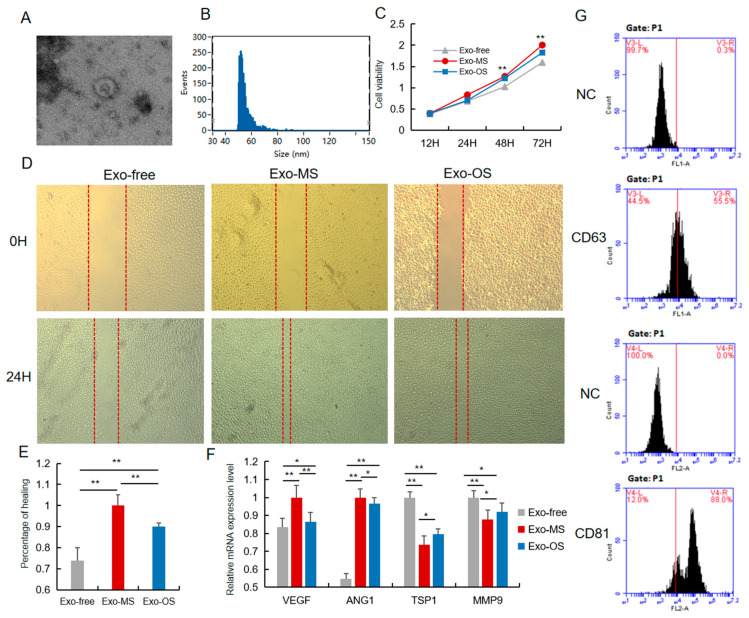
The pro-angiogenesis role of Exo-OS was repressed. (**A**) The 3D morphology of isolated pig UCB-EXO observed in electron microscope. (**B**) The line profile of electron microscope image size for UCB-EXO. X- and Y-axes are the size and events, respectively. (**C**) Cell viability was measured following Exo-OS, Exo-MS, or PBS treatment for 24 h, using the CCK8 analysis. (**D**) The migration of HUVEC cells in vitro after 24 h of treatment with Exo-MS, Exo-OS, or PBS was evaluated using scratch assay (*n* = 3). (**E**) Percentage of cells healed. (**F**) The expression level of *VEGF, ANG1, TSP1,* and *MMP9* mRNA after treating Exo-MS, Exo-OS, or PBS. (**G**) Positive rate of exosome marker proteins CD63 and CD81 detected by flow cytometry. * or ** represents significance at the 0.05 or 0.01 level, respectively.
